# Evaluation of female sexual function index and associated factors among married women in North Eastern Black Sea region of Turkey

**DOI:** 10.4274/tjod.43815

**Published:** 2014-09-15

**Authors:** Yeşim Bayoğlu Tekin, Ülkü Mete Ural, Işık Üstüner, Gülşah Balık, Emine Seda Güvendağ Güven

**Affiliations:** 1 Recep Tayyip Erdoğan University Faculty of Medicine, Department of Gynecology and Obstetrics, Rize, Turkey

**Keywords:** Sexual dysfunction, female, FSFI

## Abstract

**Objective:**

The aim of this study was detection of Female Sexual Function Index (FSFI) scores of married women living in North Eastern Black Sea region of Turkey and comparison with demographic data.

**Materials and Methods:**

A cross-sectional, descriptive study conducted at a University Hospital, gynecology and obstetrics outpatient clinic. Married women between 18-50 years of age, without any complaint enrolled in the study and participants were asked to fill out the form of FSFI. Age, gravidity and number of living children, duration of marriage, education and income levels, employment status, and contraceptive methods has been questioned. Sexual desire, arousal, lubrication, orgasm, satisfaction, pain subscales, and total score of FSFI were determined and compared with demographic data.

**Results:**

Lower FSFI levels were detected from 70.9% of the respondents. Age, duration of marriage and number of children were adversely affected the FSFI scores. Intermediate education level and usage of a contraceptive method were related with higher FSFI scores. Pain scores were high in all participants independently from other parameters.

**Conclusions:**

For identification of women’s sexual dysfunction, increasing the knowledge level and awareness about sexuality are required.

## INTRODUCTION

Sexuality is the emotional, spiritual, and behavioral interaction between two individuals, which is surrounded by cultural values, taboos, and social norms^(1)^. Human sexuality varies with culture. In our society, independent of the educational level, there is a widespread presence of sexual problems and sexual ignorance. However, the people’s refraining from, shame of, and hiding their sexuality prevent them from discussing sexual problems and getting help.

The human sexual response cycle is a four-stage model of physiological responses to sexual stimulation, which, in order of their occurrence, are the arousal phase, plateau phase, orgasm phase, and the resolution phase^(2,3)^. Men’s sexual response has one single pattern, differing only in terms of duration. On the other hand, women can have responses that differ both in intensity and duration.

In the International Classification of Diseases and Related Health Problems (ICD-10) published by The World Health Organization (WHO), sexual dysfunction is defined as the inability to fully enjoy sexual intercourse^(4)^. According to the Fourth Edition of Diagnostic and Statistical Manual of Mental Disorders published in 1999 [DSM-4], sexual dysfunction is classified as sexual desire disorder, sexual arousal disorder, orgasmic disorder, and pain related disorders^(5)^.

The management and therapy of sexual problems and sexual function impairment in men have yielded significantly good results. On the other hand, success in the diagnosis and therapy of female sexual dysfunction has been limited. One of the most important causes of this situation is that women are inhibited from expressing their sexual problems and seeking therapy due to prejudices, wrong beliefs, and sense of shame.

Studies on the female sexual dysfunction in Turkey are limited in number. However, recently, the interest in female sexual dysfunction has increased and problems related to female sexuality and sexual problems have been discussed to a greater extent than formerly. The most frequently used scale for evaluating female sexual dysfunction is the Female Sexual Function Index (FSFI). The FSFI form, which was developed by Rosen et al.^(6)^, includes 19 questions that evaluate the sexual activity. The questions are related to six topics: Sexual desire, arousal, lubrication, orgasm, satisfaction, and pain. The form has been validated for the Turkish community, and its Turkish version has been written^(7)^.

The purpose of this study was to assess the prevalence of sexual problems and the association between sexual problems and demographic variables, and some probable factors in married women living in the North Eastern Black Sea region of Turkey.

## MATERIALS AND METHODS

This investigation was a cross-sectional and descriptive study carried out in the outpatient clinic of obstetrics and gynecology of a university hospital. Married women aged 18-50 presenting for routine gynecological examination, were included in the study. Women who did not have sexual intercourse in last month, were pregnant or delivered in last 6 months or whose husband had sexual dysfunction were excluded from the study. Permission for the study was obtained from the Ethics Committee of the Faculty. Since the study was based on voluntary participation of the patients, the purpose of the study was first explained to the patients, and then those volunteering to participate were included in the study.

The participating women were first questioned on their socio-demographic features, which included age, pregnancy, number of children, duration of marriage, education, and income level. Consequently, the women were asked to fill in the FSFI form.

The scaling system of FSFI, which included questions on 6 topics, namely sexual desire, arousal, lubrication, orgasm, satisfaction, and pain, has been presented in (Table 1). The lowest score was calculated as 2 and the highest score as 36. The total FSFI score under 26.55 was accepted as sexual dysfunction^(8)^.

The data were statistically evaluated using the SPSS Statistics 17.0 package program. In the statistical analysis of the relationship between the groups, the Kruskall-Wallis test was used for the multiple independent variables (age, income level, educational level, contraceptive method), and the Mann-Whitney test was used for the two independent variables. A p value of <0.05 was accepted as statistically significant. The Pearson’s test was performed for the correlation between numerical values.

## RESULTS

A total of 175 married women of age 18-50 participated in the study. When the cut-off value for sexual dysfunction in the FSFI scale was taken as 26.55, 70.9% of the participants showed indices under the limit value. The socio-demographic features of the participants have been displayed in Table 2, and the distribution of their sexual function indices according to their socio-demographic features has been presented in (Table 3).

There was a significant relationship between the participants’ age groups and the subgroups of sexual desire (p=0.011 χ^2^= 9.021), arousal (p=0.002 χ^2^=12.207), lubrication (p=0.018 χ^2^=8.038), satisfaction (p=0.002 χ^2^=12.443) and total scores (p=0.011 χ^2^=8.974). According to these results, sexual desire in women of age 31-40 was significantly higher, and arousal, lubrication, satisfaction, and the total score in women of age 41-50 were significantly lower, than those of the other age groups. However, there was no significant difference between the age groups in terms of orgasm and pain (p=0.162 and p=0.381, respectively).

As the number of children increased, the total FSFI score decreased (p=0.049). With the increase in the duration of marriage, the total FSFI score decreased (p=0.007). The income level did not have a significant effect on the sexual function (p>0.05). When the sexual function indices of employed and unemployed women were compared, there was a significant difference in terms of lubrication (p=0.041, z=-2.042) and the total score (p=0.044, z=-2.017) (p<0.05). The values of lubrication and total score were significantly higher employed women than in unemployed women.

There was a significant difference between all the subgroups, except for pain (sexual desire: p=0.000, χ^2^= 16.981; arousal: p=0.000, χ^2^=19.455; lubrication: p=0.008, χ^2^=9.709; orgasm: p=0.000 χ^2^=18.804; satisfaction: p=0.007 χ^2^=10.008) and the total score (p=0.000, χ^2^=16.740) in terms of the educational level. The level of sexual function was higher in women with secondary school education than in women with primary school and lower education, and in women with university and higher education. However, there was no significant difference between all levels of education in terms of pain (p=0.880).

In terms of the contraception method used, there was a significant difference between arousal (p=0.048, χ^2^=9.568), orgasm (p=0.004 χ^2^=15,181), satisfaction (p=0.006 χ^2^=14.640), and the total score (p=0.020, χ^2^=11.657); however, women using no contraceptive method displayed lower scores in all subgroups. The arousal level was higher in women using hormonal contraception (p=0.023). The score of orgasm was higher in women having a contraception method than in those not using contraception (p=0.000). Sexual satisfaction scores were found to be higher in women using condom and hormonal contraception. Women using the methods of interrupted coitus and hormonal contraception had high total FSFI scores.

## DISCUSSION

The FSFI has been tested in many populations and accepted as a useful scale in screening sexual dysfunction with a cut-off value of 26.55^(8)^. The prevalence of sexual dysfunction differs based on community samples. The prevalence of sexual dysfunction determined by FSFI ranges from 43% to 69%^(9)^. A study on women of age 18-59 in the United States reported the prevalence of sexual dysfunction as 43%^(10)^. Cayan et al.^(11)^ reported this prevalence as 46.9% in Turkey. In our study, in contrast to the studies mentioned above, we determined a low sexual function index in 70.9% of the participants. The prevalence of sexual dysfunction in sexually active women was determined as 70% in Ghana by Amidu et al.^(12)^ and over 71% in Nigeria by Ojomu et al.^(13)^. Both studies reported that sexual dysfunction was associated with age, years of marriage, and number of children. Furthermore, the educational level, the working status, and use of contraceptive methods were found to have predictive values. However, no relationship was determined between the income level and sexual function^(12,13)^.

In this study, the sexual function indices of married women living in the North Eastern Black Sea region were evaluated using the FSFI scale, and their scores for each of sexual desire, arousal, lubrication, orgasm, satisfaction and pain subgroups, in addition to their total scores were calculated. According to these findings, the sexual function level was inversely affected by age and duration of marriage. It was known that menopause negatively affects the sexual functions^(14)^. In our study, we evaluated women in the reproductive ages, and although our participants were not in menopause, we observed that advancing age in women negatively affected the sexual functions. Additionally, we also determined that a long marriage life negatively affected the sexual functions. This situation may be due to the advancing age of the woman or the couple’s loss of interest for each other, or problems arising between the man and wife. Güvel et al.^(15)^ determined that the decrease in sexual function was parallel to the duration of marriage. Oniz et al.^(16)^ reported that sexual problems increased in marriages continuing for more than 11 years.

The level of sexual function was found to be higher in women with secondary school education than in women with primary school and lower education and in women with university and higher education. In the literature, the relevant data are different. Aslan et al.^(17)^ reported that sexual dysfunction was more prevalent in women with low educational level. Güvel et al.^(15)^ reported that the educational level had no effect on sexual functions, but they also stated this result could be due to the low educational level of women participating in their study. Many studies performed abroad have shown the association of low educational level with sexual dysfunction^(18,19,20)^. Studies in Nigeria^(21)^ and Malaysia^(22)^ reported that as the educational level rose, the incidence of sexual dysfunction increased. In contrast to these studies, we found higher sexual function indices in women with medium level of education. This finding can be explained by the interaction of other factors such as age, working status, and duration of marriage, with the educational level.

Our results showed that with an increase in the number of children, the sexual functions decreased (p=0.049). Likewise, Özerdoğan^(23)^ and Cayan et al.^(11)^ showed that an increase in parity negatively affected the sexual functions. As factors associated with the number of children, also the woman’s advancing age and type of delivery negatively affected the sexual functions.

Our study showed that the income level had no effect on sexual functions. It was seen that working women had higher total scores and lubrication scores. However, former studies performed in Turkey reported that the income level was closely associated with sexual functions. Özerdoğan et al.^(23)^ reported a close relationship between the income level and SFI and higher values of SFI in unemployed women. Özkan et al.^(24)^ reported that as the income increased, the sexual desire, lubrication, and satisfaction decreased, and that there was no association between sexual functions and the working status of women.

It was seen that women not practicing contraception had lower scores in all subgroups of FSFI and total scores. A study carried out in Colombia^(25)^ reported that the total FSFI score was low in women practicing natural contraception, whereas it was higher in women using modern contraception methods; however, the difference between these two groups was insignificant. İbrahim et al.^(26)^ reported that women practicing hormonal contraception and using intrauterine device had worse FSFI, whereas FSFI was not affected in women practicing no contraception. In our study, we found low FSFI scores in women practicing no contraception, which may be due to the fear of unwanted pregnancies. An interesting finding in our study was that women practicing hormonal contraception had higher indices in arousal, satisfaction, and total score. Furthermore, sexual satisfaction was found to be high in women using condom. Additionally, orgasm indices were higher in all women using contraceptive methods than in women practicing no contraception. In contrast to the former finding of no association between the contraception method and the sexual function levels in Turkey^(11)^, our study demonstrated that contraceptive methods positively affected the sexual functions of women.

In our study, we determined high pain scores in all women independent of age, duration of marriage, educational level, working status, and contraceptive method. Since dyspareunia was frequent in the participants, although none of them had expressed sexual dysfunction on presentation, and since the pain index was high independent of the demographic features, we can conclude that sexuality is still a taboo in our society. The women in our society cannot freely express their sexual problems, due to social and cultural factors and religious beliefs. The prevalence of dyspareunia in Turkey varies between 7.8% and 47.2%^(27,28)^. This high prevalence of dyspareunia, independent of the educational level may be due to absence of sexual education in schools and the low level of sexual knowledge even in women with high educational level.

Sexual life and sexual satisfaction are affected by physiological, psychological, and socio-cultural factors^(29)^. The diagnosis and determination of the prevalence of sexual dysfunction are closely associated with the methods used. The FSFI is a widely used scale for screening sexual dysfunction, but is insufficient on its own for the diagnosis of sexual dysfunction. Female sexual dysfunction is a multi-dimensional health problem caused by organic, psychological, and social factors. Anamnesis is very important in the diagnosis of sexual dysfunction. Beside FSFI, various questionnaire forms have been developed. But the greatest obstacle in front of diagnosing SFI is the refrain of the individual to express her problem as a complaint. Every woman, in whatever age or social status, presenting to the gynecologist should be questioned on sexual health and, if needed, should receive consultancy. Women should be fully informed on sexuality, so that they can express their sexuality and increase their awareness of sex.

## Figures and Tables

**Table 1:**
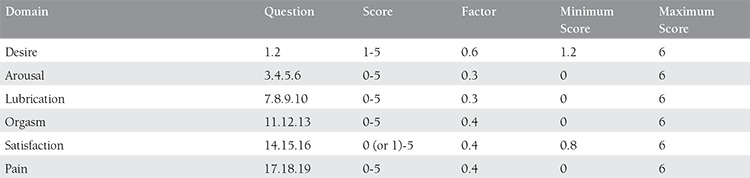
Subgroups of FSFI

**Table 2:**
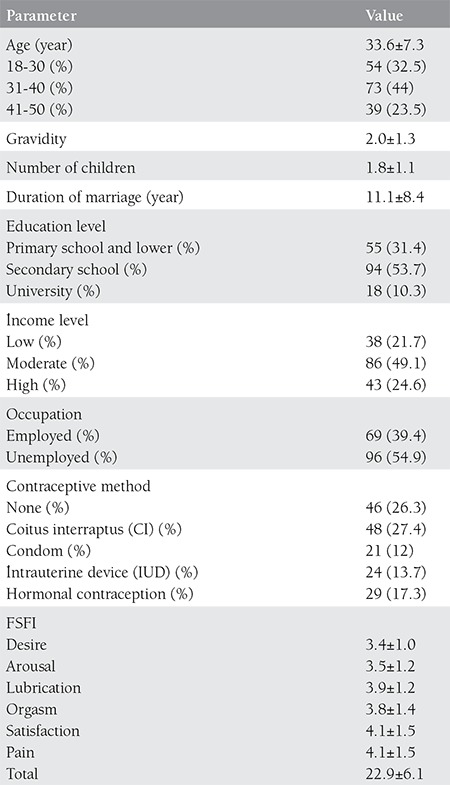
Demographic characteristics of the patients

**Table 3:**
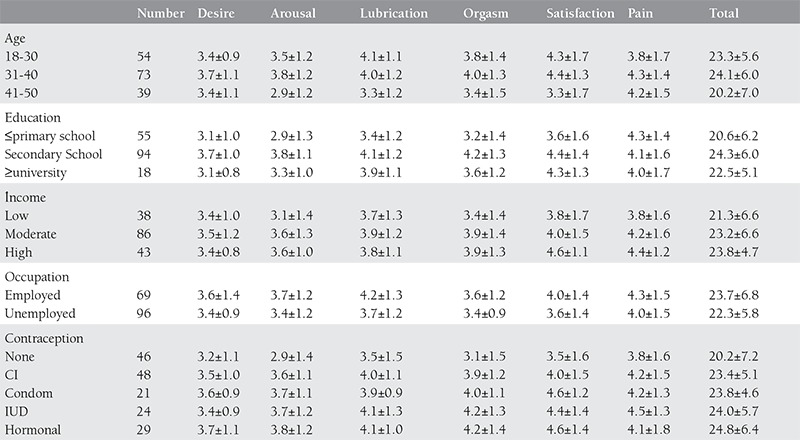
Distrubition of FSFI according to demographic characteristics
